# Low Abundances but High Growth Rates of Coastal Heterotrophic Bacteria in the Red Sea

**DOI:** 10.3389/fmicb.2018.03244

**Published:** 2019-01-07

**Authors:** Luis Silva, Maria L. Calleja, Tamara Megan Huete-Stauffer, Snjezana Ivetic, Mohd I. Ansari, Miguel Viegas, Xosé Anxelu G. Morán

**Affiliations:** Division of Biological and Environmental Sciences and Engineering, Red Sea Research Center, King Abdullah University of Science and Technology, Thuwal, Saudi Arabia

**Keywords:** Red Sea, heterotrophic bacteria, growth rates, bacterial growth efficiencies, dissolved organic matter

## Abstract

Characterized by some of the highest naturally occurring sea surface temperatures, the Red Sea remains unexplored regarding the dynamics of heterotrophic prokaryotes. Over 16 months, we used flow cytometry to characterize the abundance and growth of four physiological groups of heterotrophic bacteria: membrane-intact (*Live*), high and low nucleic acid content (HNA and LNA) and actively respiring (CTC+) cells in shallow coastal waters. Chlorophyll *a*, dissolved organic matter (DOC and DON) concentrations, and their fluorescent properties were also measured as proxies of bottom-up control. We performed short-term incubations (6 days) with the whole microbial community (Community treatment), and with the bacterial community only after removing predators by filtration (Filtered treatment). Initial bacterial abundances ranged from 1.46 to 4.80 × 10^5^ cells mL^-1^. Total specific growth rates in the Filtered treatment ranged from 0.76 to 2.02 d^-1^. *Live* and HNA cells displayed similar seasonal patterns, with higher values during late summer and fall (2.13 and 2.33 d^-1^, respectively) and lower in late spring (1.02 and 1.01 d^-1^, respectively). LNA cells were outgrown by the other physiological groups (0.33–1.08 d^-1^) while CTC+ cells (0.28–1.85 d^-1^) showed weaker seasonality. The Filtered treatment yielded higher bacterial abundances than the Community treatment in all but 2 of the incubations, and carrying capacities peaked in November 2016 (1.04 × 10^6^ cells mL^-1^), with minimum values (3.61 × 10^5^ cells mL^-1^) observed in May 2017. The high temperatures experienced from May through October 2016 (33.4 ± 0.4°C) did not constrain the growth of heterotrophic bacteria. Indeed, bacterial growth efficiencies were positively correlated with environmental temperature, reflecting the presence of more labile compounds (high DON concentrations resulting in lower C:N ratios) in summer. The overall high specific growth rates and the consistently higher carrying capacities in the Filtered treatment suggest that strong top-down control by protistan grazers was the likely cause for the low heterotrophic bacteria abundances.

## Introduction

Heterotrophic prokaryotes, bacteria and archaea (hereafter “bacteria,” since archaea are found in very low abundances in surface waters, Karner et al., 2001), represent the major living biomass in the oceans and play a vital role in marine food webs (Cotner and Biddanda, 2002; Kirchman, 2008). Their secondary production or bacterial production (BP), resulting from the consumption of labile dissolved organic matter (DOM), represents a key pathway in the transfer of matter and energy to higher trophic levels (Azam et al., 1983; Fuhrman, 1999; Kirchman et al., 2009). The amount of carbon transferred ultimately depends on the amount and quality of DOM and the composition of bacterial assemblages. In order to better understand the dynamics of marine bacteria and their interactions with DOM and other environmental variables, we can rely on community parameters such as specific growth rates (Ducklow, 2000). Focusing on carbon, once dissolved organic carbon (DOC) is assimilated, it can follow two pathways: it can be incorporated into biomass via BP or it can be remineralized through bacterial respiration (BR) (del Giorgio and Cole, 1998; Alonso-Sáez et al., 2008; Lønborg et al., 2011). Specific growth rates (μ) and bacterial biomass (BB) determine BP, which together with BR, make up the total carbon flowing through heterotrophic bacteria in natural waters (Kirchman et al., 1982; Ducklow, 2000; Kirchman, 2015). The sum of BP and BR is also known as bacterial carbon demand, BCD, which can be measured directly as the DOC being consumed in experimental incubations. Another variable relating these three is bacterial growth efficiency (BGE), which is the fraction of assimilated carbon that bacteria allocate to build up new biomass (i.e., BGE = BP/BCD). Assessments of BGE are central to understanding microbial metabolism and determining the ecological and biogeochemical role of bacteria in aquatic ecosystems, especially in the transfer of carbon to higher trophic levels (del Giorgio and Cole, 1998; Alonso-Sáez et al., 2008). However, due to uncertainties in BGE actual values across ecosystems (Briand et al., 2004; Alonso-Sáez et al., 2008; Lønborg et al., 2011), and the few seasonal studies available, the true impact of BP and BR on the marine carbon cycle is still hard to predict.

Among all the possible factors affecting heterotrophic bacterioplankton abundance and productivity, research has usually focused on the following: resource availability (inorganic and organic nutrient and carbon sources) or bottom-up control (Bird and Kalff, 1984; Ducklow and Carlson, 1992), mortality due to protistan grazing and viral lysis or top-down control (Thingstad and Lignell, 1997; Jürgens and Massana, 2008; Vaqué et al., 2014) and water temperature (White et al., 1991; Morán et al., 2010). Other potentially important physico-chemical factors include light (Church et al., 2004; Ruiz-González et al., 2012) or salinity (Griffiths et al., 1984). There have been attempts in controlled experiments to isolate bottom-up (e.g., Wetz and Wheeler, 2004) from top-down processes (e.g., Longnecker et al., 2010), but both types of control occur simultaneously in the field. Environmental conditions can impact their relative influence (Thingstad, 2000). Recent efforts at understanding the joint effect of bottom-up vs. top-down controls and temperature on heterotrophic bacteria (Morán et al., 2017, 2018) predict when and where will temperature become prevalent. However, there are still gaps in our knowledge about the relative importance of bottom-up and top-down processes in determining heterotrophic bacterial abundance and production from a temporal perspective, especially in oligotrophic environments (Fuhrman and Hagström, 2008; Jürgens and Massana, 2008; Morán et al., 2018). More generally, basic knowledge about the temporal dynamics of heterotrophic bacteria in subtropical and tropical waters is still scarce when compared with the usually richer, higher latitude environments. A handful of long-term programs have been developed to fill some of these gaps, such as the Bermuda Atlantic Time Series (BATS) in the Sargasso Sea at the western North Atlantic Subtropical Gyre (Steinberg et al., 2001), the Hawaii Ocean Time Series (HOT) situated in the North Pacific Subtropical Gyre (Karner et al., 2001; Karl and Church, 2014), and the San Pedro Ocean time-series (SPOT) off the coast of California (Cram et al., 2015). These studies show that even with apparently more stable conditions, seasonal changes in bacterioplankton community composition in subtropical and tropical oceans were similarly linked to environmental drivers (Bunse and Pinhassi, 2017).

One of such largely underexplored subtropical and tropical areas is the Red Sea. The Red Sea is also one of the youngest semi-enclosed marine basins of the world, characterized by some of the warmest temperatures (22 to 35°C at the surface, Rasul et al., 2015; Chaidez et al., 2017) and the highest salinities (Tesfamichael and Pauly, 2016). For those reasons, although similar in many aspects to the large subtropical gyres, the oligotrophic Red Sea could be used as a model of the future ocean conditions elsewhere. Pioneering attempts at describing bacterioplankton dynamics were mostly restricted to the northernmost and southernmost regions (Weisse, 1989; Wiebinga et al., 1997; Grossart and Simon, 2002). More recent studies have linked the structure and composition of microbial communities with environmental gradients (Ngugi et al., 2012; Pearman et al., 2017; Thompson et al., 2017), and given insights into the distribution of microbial plankton within the mixed layer (Calbet et al., 2015). However, information about the dynamics of Red Sea heterotrophic bacterial standing stocks is still very limited.

Preliminary information about central Red Sea epipelagic waters (Calleja et al., 2018; García et al., 2018) suggest that the abundances of heterotrophic bacteria could be notably lower than the mean surface values of the world ocean (Arístegui et al., 2009). Here, we assessed the influence of environmental factors, focusing on temperature and bottom-up controls, including direct (DOM and inorganic nutrients concentrations) and indirect variables (chlorophyll *a*), on the specific growth rates and carrying capacities of four different physiological groups (del Giorgio and Gasol, 2008) of marine bacteria in a coastal ecosystem in the central Red Sea. Specifically, high and low nucleic acid content, membrane-intact and actively respiring cells were analyzed by flow cytometry. For over a year, monthly short-term incubations with and without the presence of protistan grazers were performed, allowing us to also advance our knowledge about the relative importance of bottom-up and top-down controls in these tropical shallow waters. The objectives of this study were: (i) to assess the seasonal variability of the growth rates and carrying capacities of four physiological groups of heterotrophic bacteria and (ii) to test the role of DOM and temperature on bacterial carbon usage. We hypothesized that the low abundances of heterotrophic bacteria in central Red Sea waters would be to some extent constrained by protistan grazing while bacterial activity should reflect changes in DOM concentration and quality.

## Materials and Methods

### Sampling Site and Experimental Design

Surface waters were collected from the harbor of King Abdullah University of Science and Technology (KAUST) (22°18′23.20″ N, 39°6′10.71″ E). Incubations were conducted monthly over the course of 16 months (December 2015 until March 2017). Ancillary physico-chemical variables, including temperature [°C] and salinity were measured at the surface immediately prior to sampling with an environmental probe (YSI probe). Water sampling was performed using a pre-clean (acid-washed) polycarbonate 9 L carboy.

The site was characterized monthly by following changes in the above-mentioned physico-chemical variables plus total chlorophyll a (chl *a*). Inorganic nutrient concentration, DOC and DON concentrations, DOM fluorescence, bacterial abundance and bacterial single–cell physiological properties were also measured at the starting time of incubations.

Experimental incubations of 4–6 days were then performed to follow bacterial and DOM dynamics in two different conditions: with unfiltered water to asses the dynamics of the entire microbial community; and with filtered water (through pre-combusted Whatman GF/C filters, 1.2 μm nominal size) to target the interaction between heterotrophic bacteria and DOM after removing protistan grazers. The experimental incubations were performed in triplicates (2 L bottles) and conducted using the seawater culture method (Ammerman et al., 1984) in temperature-controlled incubators (Percival – I-22LLVL) with *in situ* light (12 h light/12 h dark cycle) and temperature conditions. Samples of inorganic nutrients, DOC, DOM fluorescence, bacterial abundance and bacterial single – cell physiological properties were collected daily during the incubations, except for bacterial abundance that, based on previous tests, were collected twice per day until the abundance of bacteria reached a plateau or started to drop (usually after 1 or 2 days). Incubations lasted not longer than 6 days, once the stationary or decay phase of bacterioplankton growth was well observed. We did not consider secondary growth phases for calculations.

Concentrations of size-fractionated chlorophyll *a* (chl *a*) were obtained after filtration of 200 ml samples through polycarbonates filters of 20, 2, and 0.2 μm. Filters were kept in the -80°C Freezer until analysis. Chl *a* were extracted in 90% acetone for 24 h in the dark at 4°C and measured with a Turner model Trilogy Fluorometer using the acidification method calibrated with chlorophyll standard (Anacystis nidulans, Sigma Aldrich). Total chl *a* was calculated with summing of the three fractions.

### Inorganic Nutrients and Dissolved Organic Carbon (DOC) and Nitrogen (DON)

Samples for nutrient analyses were also filtered through 0.2 μm Millipore^®^ polycarbonate filters and stored frozen at -20°C until analysis. Nitrate (NO_3_^-^), nitrite (NO_2_^-^), and phosphate (PO_4_^3-^) were analyzed in a segmented flow analyzer from Seal Analytical. All standards were prepared with a nutrient-free artificial seawater matrix in acid-washed glassware. Consumption and production rates of inorganic nutrients (in μmol L^-1^ d^-1^) were estimated during the exponential growth period as the difference between the initial and final concentration divided by time. Positive and negative values were considered as production and consumption, respectively.

Samples for DOC and total dissolved nitrogen (TDN) were filtered through 0.2 μm Millipore^®^ polycarbonate filters. After the filtration, each sample was acidified with H3PO4 and kept at 4°C until further analysis by high temperature catalytic oxidation (HTCO) using a Shimadzu TOC-L. Reference material of deep-sea carbon (42–45 μmol C L^-1^ and 31–33 μmol N L^-1^) and low carbon water (1–2 μmol C L^-1^) was used to monitor the accuracy of DOC and TDN concentration measurements. DON concentrations were calculated after subtracting the dissolved inorganic nitrogen (DIN) to the TDN (DON = TDN-DIN), where DIN (μmol C L^-1^) = [NO_3_^-^]+ [NO_2_^-^].

### DOM Fluorescence Measurements

UV-VIS fluorescence spectroscopy was measured using a HORIBA Jobin Yvon AquaLog spectrofluorometer with a 1 cm path length quartz cuvette. Three dimensional fluorescence excitation emission matrices (EEMs) were recorded by scanning the excitation range between 240 and 600 nm and emission wavelength range of 250–600 nm, both at 3 nm increments and using an integration time of 8 s. To correct and calibrate the fluorescence spectra post-processing steps were followed according to Murphy et al. (2010). Briefly, Raman-normalized Milli-Q blanks were subtracted to remove the Raman scattering signal (Stedmon et al., 2003). All fluorescence spectra were Raman area (RA) normalized by subtracting daily blanks that were performed using Ultra-Pure Milli-Q sealed water (Certified Reference, Starna Cells). Inner-filter correction (IFC) was also applied according to McKnight et al. (2001) RA normalization, blank subtraction, IFC and generation of EEMs were performed using MATLAB (version R2015b). The EEMs obtained were subjected to PARAFAC modeling using drEEM Toolbox (Murphy et al., 2013). Before the analysis, Rayleigh scatter bands [first order at each wavelength pair where Ex = Em ± bandwidth; second order at each wavelength pair where Em = 2 Ex ± (2 × bandwidth)] were trimmed. No samples were identified as outliers and the model was validated using split-half validation and random initialization (Stedmon and Bro, 2008). A four-component model was validated. Peak C1 at Ex/Em 240(321)/417 nm, peak C2 at Ex/Em 255(369)/467 nm, peak C3 at Ex/Em 240(291)/342 and peak C4 at Ex/Em 273/312 nm. The maximum fluorescence (Fmax) is reported in Raman units (RU) (Stedmon et al., 2003; Murphy et al., 2010). Consumption and production rates from each of the DOM fluorescent components (ΔRU, RU d^-1^) were estimated as the decrease in total fluorescence (RU_initial_–RU_max/min_) during the exponential growth phase. Positive and negative values were considered as production and consumption, respectively.

### Single-Cell Physiological Groups of Heterotrophic Bacteria and Cyanobacteria

Five different single-cell groups of heterotrophic bacteria were considered in order to describe the physiological structure (del Giorgio and Gasol, 2008) of the site. We followed the methodology described in more detail in Gasol and Morán (2015). High nucleic acid (HNA) and low nucleic (LNA) cells were distinguished by their green fluorescence signal after being stained with SYBR Green (Marie et al., 1997). Samples for these two groups were previously fixed with 1% paraformaldehyde + 0.05 mL glutaraldehyde (final concentration) and deep-frozen in liquid nitrogen and stored at -80°C until analysis. Usually within 1 and 3 months, samples were thawed, stained and run in a BD FACSCanto flow cytometer. Cyanobacteria of the genus *Synechococcus* were also counted in the same samples, easily differentiated by their higher red fluorescence signal due to chlorophyll *a* and orange fluorescence due to phycoerythrin.

Cells with intact membranes (*Live*) were distinguished from membrane-compromised cells (*Dead*) by combining two nucleic acid stains, SYBR Green (Molecular Probes) and Propidium Iodide (Sigma Chemical Co.) (Grégori et al., 2001). Differently to the HNA/LNA group, *Live* and *Dead* cells were analyzed without prior fixation.

Actively respiring cells (CTC+) were distinguished by the red fluorescence signal that indicates the deposition of oxidized crystals of the CTC-tetrazolium salt (Sherr et al., 1999). This group was also analyzed *in vivo* after an incubation period of 90 min in the dark after with the CTC-tetrazolium salt.

### Bacterial Abundance and Biomass

The abundances of the five physiological groups were calculated after gravimetric calibration of the flow rates. Total bacterial abundances correspond to the sum of LNA and HNA cells described in the previous section. Filtration by GF/C filters resulted in an average loss of 19% of the cells present in the Community treatment.

Latex fluorescent beads of 1 μm diameter (Molecular Probes) were added to all samples as an internal standard. Side scatter (SSC, light scatter at 90°) values of the HNA and LNA groups relative that of the fluorescent beads was converted to cell diameter following the empirical calibration of Calvo-Díaz and Morán (2006). Cell diameter was converted to volume and finally converted into bacterial biomass (BB) using the estimation from Gundersen et al. (2002): fgC cell^-1^ = 108.8 × [Bv]^0.898^ and then converted into μmol C L^-1^, where the Bv corresponds to the biovolume.

We also estimated the abundance of picophytoplankton in the Filtered treatment in all our cytograms. The only group of autotrophic organisms found in the GF/C filtrate was *Synechococcus* (*Prochlorococcus* was only present 4 times in our weekly sampling of this shallow site since June 2015). Their abundances were on average 86% lower (ranging from 33 to 97%) than those in the unfiltered water (Community treatment).

### Growth Rates and Carrying Capacities

The growth rates (μ) of HNA, LNA, *Live* and CTC+ cells were calculated for both treatments as the slope of the natural logarithm of bacterial abundances vs. time during the corresponding exponential growth phases. Due to the short-time of the exponential growth periods (usually 1–2 days), viruses were unlikely to have affected bacterial responses. For that treason, we will use the term “net growth rate” for the Community treatment while “specific growth rate” will be used for the Filtered treatment.

Carrying capacities were estimated as the maximum abundances recorded for each bacterial group at the plateau stage of the incubations.

### Bacterial Growth Efficiency

Bacterial growth efficiencies (BGE, %) were estimated by following changes in DOC concentrations and bacterial biomass over time, as shown in the following formula: BGE (%) = [ΔBB/-ΔDOC] × 100, where, ΔBB represents the increase of total bacterial biomass (BB_max_-BB_initial_, in μmol C L^-1^ d^-1^), and ΔDOC is the carbon consumption (μmol C L^-1^ d^-1^) estimated from the decrease in total DOC (DOC_initial_-DOC_min_). Estimations of ΔBB and ΔDOC were done during the exponential growth phase using the same period of time for both components. We assume that the remaining C (100%-BGE) corresponds to bacterial respiration.

Although *Synechococcus* initial abundances were low, sometimes they grew during the incubations. We estimated DOC production by growing *Synechococcus* cells for the same period for which we calculated BGE (i.e., the exponential phase of bacterial growth). The relative side scatter flow cytometric signal was first converted into cell volume following the empirical calibration of Calvo-Díaz and Morán (2006) and then into cell-specific biomass using 230 fg C μm^3^ (Worden et al., 2004). We used the changes in abundance and cell-specific biomass to calculate *Synechococcus* biomass increase (μmol C L^-1^ d^-1^). Since this increase in *Synechococcus* biomass corresponded to particulate primary production (PPP), we subsequently estimated the dissolved primary production (DPP) potentially contributing to DOC fluxes. For that, we assumed a very conservative value of 50% percent extracellular release (PER) [PER = DPP/(DPP + PPP)], much higher than the expected PER for exponentially growing phytoplankton cells (e.g., Morán et al., 2002; Huete-Stauffer et al., 2017). Even with this unrealistically high DPP values, this input would only represent on average 4.8% of the changes in DOC during the exponential phase of bacterial growth. The measurements of BGE barely changed, and definitely the seasonal pattern depicted was not altered.

### Data Analysis

JMP PRO 13 software was used for statistical analyses. One-way analyses of variance (ANOVA) and *post hoc* Tukey HSD tests were used to identify significant (*p* < 0.05) differences between groups or treatments. Pearson r coefficients are given for correlation analyses.

## Results

### Initial Conditions

**FIGURE 1:**
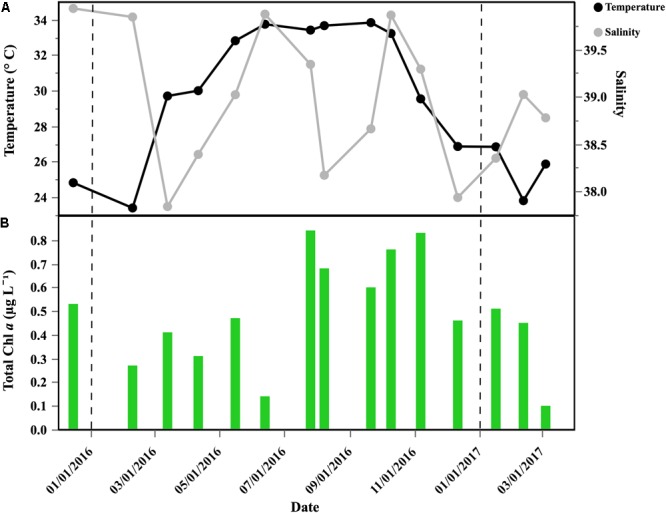
Temporal distribution of selected environmental variables in the coastal sampled site of the central Red Sea: **(A)** temperature (°C) and salinity; **(B)** Chl *a* concentration (μg L^-1^). Vertical dash black lines represent the beginning of a new year.

High surface temperatures were found year-round (Figure 1A), yet showing a clear seasonality with minimum values in February (23.4°C) and maximum in September (33.8°C). We observed high and constant temperatures from May 2016 to October 2016 (average 33.4 ± 0.4°C), and temperature started to decrease again from September 2016 onward. Salinity changes were less marked, with lower values (ca. 37) recorded in late February 2016 and April 2016, and higher values (ca. 40) frequently observed from mid-July to October 2016 (Figure 1A). Chl *a* ranged from 0.10 to 0.84 μg L^-1^, peaking during July and November, with minimum values in March 2017 (Figure 1B).

Inorganic and organic nutrient concentrations at the onset of the incubations are shown in Table 1. The concentration of nitrate ranged widely from 2.2 to 28.8 μmol L^-1^, with an extremely high concentration (83.7 μmol L^-1^) in October 2016. Phosphate concentrations ranged from 0.038 to 0.115 μmol L^-1^ (Table 1).

**Table 1:** Initial concentrations of nitrate, phosphate, DOC, DON, DOM C:N ratios^1^, and fluorescence intensity of the identified DOM fluorescent components for the incubations.

Day	Temperature (°C)	Nitrate (μmol L^-^^1^)	Phosphate (μmol L^-^^1^)	DOC (μmol L^-^^1^)	DON (μmol L^-^^1^)	C:N ratio	C1 (RU)	C2 (RU)	C3 (RU)	C4 (RU)
12/15/15	26.8	3.5	0.082	80.8	4.8	16.8	0.036	0.018	0.025	0.024
2/9/16	23.4	2.2	0.039	98.2	5.9	16.6	0.028	0.015	0.018	0.017
3/13/16	29.7	5.0	0.085	83.7	5.2	16.1	0.032	0.026	0.012	0.028
4/11/16	30.0	5.9	0.041	90.6	8.2	11.0	0.039	0.022	0.028	0.019
5/16/16	32.8	3.0	0.045	89.3	8.8	10.1	0.040	0.021	0.032	0.013
6/13/16	33.7	21.2	n.a	88.6	10.3	8.6	0.054	0.027	0.020	0.011
7/26/16	33.4	28.8	0.099	86.9	7.4	11.7	0.064	0.032	0.026	0.013
8/8/16	33.7	23.2	0.085	86.5	10.1	8.6	0.049	0.026	0.019	0.010
9/21/16	33.8	18.0	0.045	89.3	5.9	15.1	0.050	0.027	0.021	0.011
10/10/16	33.2	83.7	0.071	91.9	4.2	21.9	0.083	0.037	0.043	0.012
11/7/16	29.5	9.9	0.038	87.5	6.5	13.5	0.054	0.029	0.028	0.011
12/12/16	26.9	6.2	0.084	85.2	3.5	24.3	0.044	0.024	0.051	0.007
1/17/17	24.8	3.9	0.078	73.8	2.5	29.5	0.021	0.013	0.016	0.009
2/13/17	23.8	5.2	0.091	76.4	4.3	17.8	0.037	0.020	0.020	0.009
3/5/17	25.9	3.3	0.115	74.1	8.3	8.9	0.030	0.016	0.017	0.012

*^*1*^C:N ratios were obtained by dividing DOC by DON.*

Dissolved organic carbon concentration ranged from 73.8 to 98.2 μmol C L^-1^, and with the exception of the peak in February, it showed consistently higher concentrations from April through October 2016 (average 88.8 ± 1.8 μmol C L^-1^). From October 2016 onward, DOC concentrations decreased consistently. DON ranged from 2.5 to 10.1 μmol N L^-1^ and a clear seasonality was observed, with an increase from December 2015 to June 2016 followed by a continuous decrease from August 2016 to January 2017. DON increased significantly with temperature (*r* = 0.52, *p* = 0.04, *n* = 15). C:N ratios of DOM averaged 15.4 ± 6.2 and showed an inverse seasonal pattern of DON, with minimum values found between April and August 2016 (Table 1). The C1 fluorescent component of DOM, with a humic-like nature and corresponding to peak M in Coble (2007), presented higher fluorescent intensity than the other components throughout the year (ANOVA, *p* < 0.001, *n* = 15). The tyrosine-like C4 component showed consistently lower values (i.e., C1 > C2 = C3 > C4, Tukey HSD, *p* = 0.01 and *p* = 0.004, *n* = 15, for C2 and C3, respectively) (Table 1).

**FIGURE 2:**
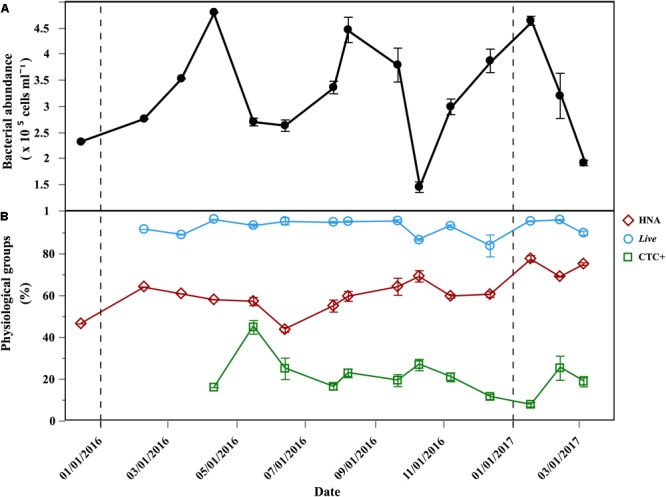
Temporal distribution of bacterioplankton physiological groups at the beginning of the incubations. **(A)** Average (±SE) of initial total abundance (HNA + LNA) in the Community treatment; **(B)** average (±SE) percentages of: HNA, *Live* cells, CTC+ cells, in the Community treatment; vertical dash black lines represent the beginning of a new year.

Total bacterial abundance ranged from 1.46 to 4.81 × 10^5^ cells mL^-1^ (Figure 2A) and HNA cells dominated for most of the year, with percentages ranging from 42.6 to 77.6% (Figure 2B). *Live* cells dominated year-round and averaged 92.8 ± 4.6% (Figure 2B). The initial abundances of *Live* cells ranged from of 1.37 × 10^5^ cells mL^-1^ to 4.75 × 10^5^ cells mL^-1^ (Supplementary Table S1). Actively respiring cells (CTC+) were consistently one order of magnitude less abundant than *Live* cells. The initial abundances of CTC+ cells varied from 3.64 to of 12.1 × 10^4^ cells mL^-1^ (Supplementary Table S1), with an average contribution of 21 ± 8.8%, although it varied widely (Figure 2B). Initial abundances for the Filtered treatment are shown in the Supplementary Table S1.

### Growth Rates and Carrying Capacities

**FIGURE 3:**
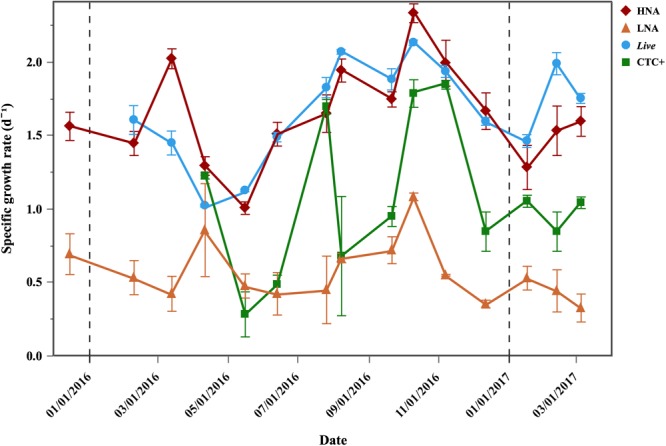
Temporal distribution of average (±SE) specific growth rates of bacterial physiological groups in the Filtered treatment. Vertical dash black lines represent the beginning of a new year.

Although net growth rates were positive year-round in the Community treatment (0.32–1.46 d^-1^), values were consistently higher in the Filtered treatment (paired *t*-test, *p* < 0.001, *n* = 42), ranging from 0.79 to 2.02 d^-1^. Figure 3 shows the values of the 4 physiological groups (*Dead* cells excluded) in the Filtered treatment. *Live* and HNA specific growth rates had similar patterns (*r* = 0.63, *p* < 0.001, *n* = 42), with increases from May onward, reaching a common peak in October (2.13 and 2.33 d^-1^, respectively) and respective minima of 1.02 and 1.01 d^-1^ in May and April, respectively (Figure 3). A secondary peak was also observed in March for the HNA cells (1.97 d^-1^) and in February for *Live* (1.99 d^-1^). LNA cells also showed an increase in specific growth rates from summer to fall, reaching a maximum in October 2016 (1.08 d^-1^, Figure 3). LNA cells were consistently outgrown by the other groups, showing significantly lower specific growth rates than HNA, *Live* and CTC+ cells (ANOVA, *p* < 0.0001, *n* = 45, *n* = 42, *n* = 36, for HNA, *Live* and CTC+, respectively). As for HNA and *Live* cells, CTC+ cells specific growth rates also peaked in October 2016 (1.79 d^-1^), but overall they showed significantly lower values than HNA and *Live* cells (i.e., μ HNA = *Live* > CTC+ > LNA, Tukey HSD, *p* < 0.001, *n* = 36) and a less obvious seasonal pattern (Figure 3).

**FIGURE 4:**
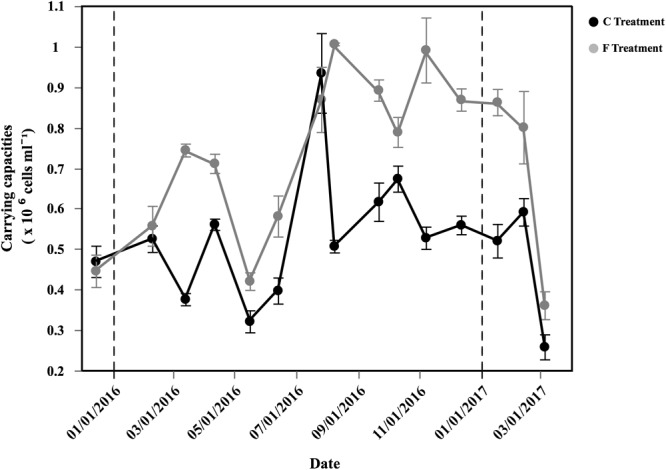
Temporal distribution of average (±SE) carrying capacities of total bacteria (HNA + LNA) in the two different treatments.

The carrying capacities of the entire bacterial assemblage showed clear seasonal patterns in both the Community and Filtered treatments (Figure 4), resemble the variability in specific growth rates of HNA and *Live* cells (Figure 3). In the Filtered treatment, carrying capacities reached 1.04 × 10^6^ cells mL^-1^ in August 2016 and minimum values in May 2016 (3.61 × 10^5^ cells mL^-1^, Figure 4). Likewise specific growth rates, HNA and *Live* cells carrying capacities had similar patterns, with high values from summer until winter (Supplementary Table S1). Carrying capacities of HNA and CTC+ cells reached abundances of 8.93 and 1.71 × 10^5^ cells mL^-1^, respectively, while *Live* cells reached a maximum of 9.72 × 10^5^ cells mL^-1^ and LNA cells peaked at 2.37 × 10^5^ cells mL^-1^ (Supplementary Table S1). The corresponding carrying capacities were positively correlated with the initial abundance for the total (*r* = 0.66, *p* < 0.001, *n* = 40), HNA (*r* = 0.58, *p* < 0.001, *n* = 40) and LNA cells (*r* = 0.76, *p* < 0.001, *n* = 40).

The Filtered treatment consistently yielded higher carrying capacities values than the Community treatment except in December 2015 and July 2016 (paired *t*-test, *p* < 0.001, *n* = 45). The ratio between the Filtered and the Community treatment carrying capacities, a proxy of top-down control, ranged between 0.93 and 1.98 (Supplementary Table S2).

### DOM Consumption Rates and Bacterial Growth Efficiencies

Estimation of DOC consumption rates (ΔDOC/Δt) was only possible in the Filtered treatment because a net increase in DOC was frequently observed in the Community treatment, most probably due to DOC production by phytoplankton and other planktonic groups present in the samples. ΔDOC/Δt ranged from 1.33 to 10.6 μmol C L^-1^ d^-1^ (Table 2). A decrease in DOC consumption rate was observed from February to May 2016, followed by an increase until fall. The percentage of DOC consumed daily (%DOC) during the exponential phase of bacterial growth, an indication of its lability, ranged from 1.5 to 10.8% (Table 2). Both ΔDOC/Δt (*r* = -0.53, *p* = 0.04, *n* = 15) and %DOC/Δt (*r* = -0.60, *p* = 0.02, *n* = 13) were negatively correlated with environmental temperature.

**Table 2:** Date, DOC consumption rates (ΔDOC/Δt), bacterial biomass increase (ΔBB/Δt), bacterial growth efficiency (BGE), percentage of DOC consumed and consumption (<0) or production (>0) rates of inorganic phosphate (ΔPhosphate/Δt) in the incubations.

Day	ΔDOC/Δt (μmol C L^-^^1^ d^-^^1^)	ΔBB/Δt (μmol C L^1^ d^-^^1^)	BGE (%)	DOC consumed (% d^-^^1^)	ΔPhosphate/Δt (μmol L^-^^1^ d^-^^1^)
12/15/15	8.70	0.23	2.7	10.8	–0.050
2/9/16	10.60	0.27	2.5	10.8	–0.029
3/13/16	4.04	0.30	7.4	4.8	–0.007
4/11/16	6.37	0.30	4.7	7.0	0.094
5/16/16	1.33	0.17	12.6	1.5	0.014
6/13/16	1.68	0.22	13.0	1.9	n.a
7/26/16	2.39	0.23	9.5	2.8	–0.046
8/8/16	6.54	0.49	7.6	7.6	–0.031
9/21/16	6.14	0.39	6.4	6.9	–0.014
10/10/16	8.85	0.63	7.2	n.a	–0.018
11/7/16	5.36	0.47	8.7	6.1	–0.002
12/12/16	7.86	0.42	5.3	9.2	–0.009
1/17/17	6.09	0.31	5.1	8.3	–0.025
2/12/17	7.73	0.24	3.1	10.1	0.005
3/5/17	3.12	0.13	4.0	4.2	–0.028

**FIGURE 5:**
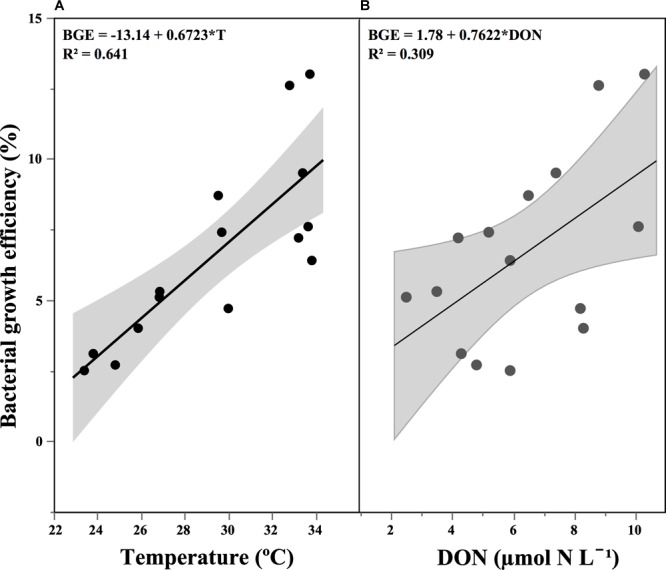
Relationship between bacterial growth efficiency and: **(A)** temperature and **(B)** dissolved organic nitrogen concentration (DON). The shaded area represents the confidence intervals (95%) for the fitted linear regression.

The rates of heterotrophic bacterial biomass increase (ΔBB/Δt), concurrent to the DOC consumption rates detailed above, ranged from 0.13 to 0.63 μmol C L^-1^ d^-1^ (Table 2), reflecting largely the changes in specific growth rates (Figure 3). Bacterial growth efficiency [i.e., (ΔBB/Δt)/(ΔDOC/Δt)] ranged from 2.5 to 13.0% (Table 2). We observed a relative increase during spring, which was followed by a slow decrease until late fall, except for a secondary peak in November. BGE values kept decreasing during winter (Table 2). We observed a positive correlation of BGE with *in situ* temperature (*r* = 0.80, *p* < 0.001, *n* = 15, Figure 5A) and DON, *p* = 0.03, *n* = 14, Figure 5B).

**FIGURE 6:**
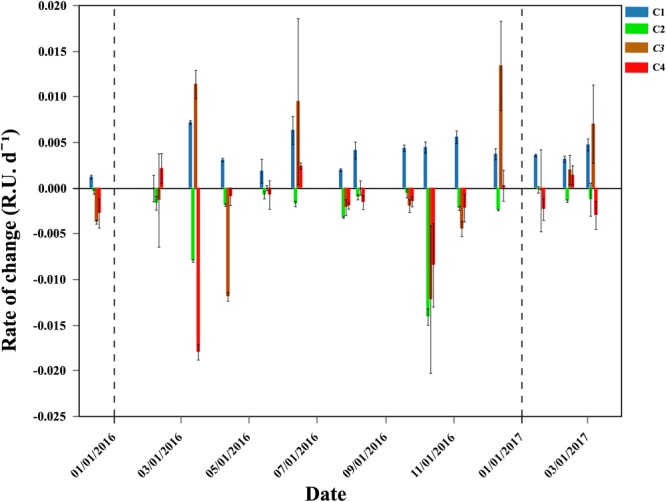
Temporal distribution of average (±SE) production (>0) or consumption (<0) rates (RU d^-1^) of the four DOM fluorescent components. Vertical dash black lines represent the beginning of a new year.

The DOM fluorescent components C2, C3, and C4 showed consistent consumption during the exponential phase of bacterial growth, with maximum values of 0.0141, 0.0119, and 0.0180 RU d^-1^ and minimum 0.0002, 0.0003, and 0.0008 RU d^-1^, respectively (Figure 6). The consumption rates of these 3 components peaked around the same period, with maxima for C2 and C4 observed in October 2016. C3 consumption rates also peaked in October but higher consumptions rates were also measured earlier in the year (Figure 6). However, C3 behaved quite erratically within the incubations, so the consumption rates observed (with high associated standard deviations) should be taken with caution. Contrary to the other FDOM components, the humic component C1 was consistently produced during the incubations, ranging from 0 to 0.007 RU d^-1^ (Figure 6). *In situ* values and the rate of change observed in the incubations identified C1 as refractory humic DOM material. On the contrary, C2, which also showed a humic-nature, decreased in all incubations. C4 represented the more labile protein-like material, with low *in situ* values (Table 1) suggesting high turnover rates. Its lability was further evidenced by being consumed in almost all the incubations until exhausted.

The specific growth rates of HNA and CTC+ cells were negatively correlated with the rate of change of the component C4 (i.e., the higher the consumption the higher the μ values, *r* = -0.56, *p* = 0.029, *n* = 15 and *r* = -0.64, *p* = 0.024, *n* = 12, respectively). The growth rates of HNA were also negatively correlated with the rate of change of the component C2 (*r* = -0.74, *p* = 0.002, *n* = 15) and the CTC+ cells positively correlated with initial total Chl *a* values (*r* = 0.60, *p* = 0.04, *n* = 12).

Consumption of phosphate was also observed for most of the year in the Filtered treatment, with rates ranging between 0.002 and 0.110 μmol L^-1^ d^-1^ (Table 2). Assuming a C:P ratio of 35 for growing bacteria (Vrede et al., 2002), we estimated that phosphate consumption was enough to fulfill bacterial P requirements in 7 months, and averaged 57% in the remaining ones. In contrast, inorganic nitrogen (nitrate and nitrite) was only occasionally taken up in the experiments (data not shown).

## Discussion

The role of heterotrophic bacterioplankton in the oceans is predicted to increase under warmer conditions (Sarmento et al., 2010; Morán et al., 2015). The Red Sea, due to its already warm temperatures throughout the water column (ca. 22°C down to the bottom, Rasul et al., 2015), could be used as a model for future, warmer ocean scenarios. However, except for the Gulf of Aden and Aqaba (Weisse, 1989; Grossart and Simon, 2002), knowledge on bacterioplankton dynamics in the Red Sea is still poor. Here, we present the first systematic report on the seasonal variability of heterotrophic bacterioplankton growth rates and efficiencies in Red Sea waters, adding to the limited knowledge of the biogeochemical role of heterotrophic bacteria in tropical ecosystems.

With a mean annual value slightly higher than 2 × 10^5^ cells mL^-1^, heterotrophic bacterial abundances measured in our study were substantially lower than average surface values of the world ocean reviewed by Arístegui et al. (2009). Previous studies in the central Red Sea have shown higher abundances, ranging from around 0.5 to 1.5 × 10^6^ cells ml^-1^ (Weisse, 1989; Calbet et al., 2015; Kürten et al., 2015). Our abundances were also lower than those measured in the northernmost and southernmost regions (Gulfs of Aqaba and Aden), where abundances higher than 5 × 10^5^ cells ml^-1^ were also frequently recorded (Wiebinga et al., 1997; Grossart and Simon, 2002; Kürten et al., 2015). Other tropical systems, with similar chl *a* values, also tend to show higher abundances. For instance, 3 to 7 × 10^5^ cells ml^-1^ were found in the Sargasso Sea (Carlson et al., 1996; Gundersen et al., 2001), while abundances >1 × 10^6^ cells ml^-1^ were frequent in the Atlantic Meridional Transect (Hale et al., 2017) and in the San Pedro Ocean Time Series (Cram et al., 2015) The lower abundances reported here could be explained by the different methodological approaches used in earlier studies in the Red Sea [i.e., microscopy in Wiebinga et al. (1997) and Grossart and Simon (2002)], inter-annual differences or by strong top-down control. Indeed, the high specific growth rates (cf. with a similar study in a temperate latitude, Huete-Stauffer et al., 2015) and the fact that the carrying capacities were indeed closer to the environmental abundances described for nearby Red Sea locations also using flow cytometry (e.g., Calbet et al., 2015; Kürten et al., 2015), suggest that protistan grazers were actively precluding the accumulation of heterotrophic bacterioplankton at our shallow coastal site. Interestingly, in all but 2 of 12 experiments conducted in the southern Bay of Biscay the carrying capacities were higher in the Community treatment (Huete-Stauffer et al., unpublished data), with a similar variability in net and specific growth rates (Morán et al., 2018), while we found just the opposite (Figure 4). Contrary to the Red Sea, nutrient availability and temperature in that temperate ecosystem were the alternating controlling factors rather than top-down control (Calvo-Díaz et al., 2014). We interpret our general higher carrying capacity ratios between the Filtered and the Community treatments as evidence that removing protistan grazers was more important than reducing the additional supply of DOC provided by the rest of the microbial community retained by the filters.

In addition to our lower initial bacterial abundances, the relatively low carrying capacities (annual mean 7.31 × 10^5^ cells ml^-1^) in the treatment without grazers suggests there is some other limiting factor precluding heterotrophic bacterioplankton reaching high abundances in spite of the relatively high DOC concentrations that were occasionally found (Table 1). Although the potential for growth existed, the standing stocks were surprisingly low, and likely kept at those values by strong top-down control. The significantly higher carrying capacities in the Filtered treatment also hint that top-down control exerted by protistan grazers was strong year-round in our system, especially in the second half of 2016. In a large survey in the tropical and subtropical ocean, either strong bottom-up or top-down control at low latitudes are hypothesized to prevent large increases in standing stocks in response to higher temperatures (Morán et al., 2017).

In the few early studies conducted in the Red Sea, specific growth rates of heterotrophic bacteria varied widely. In the northern Red Sea, a maximum value of 0.15 d^-1^ was measured by Grossart and Simon (2002), while in the central and southern Red Sea, averages of 1.5 ± 0.6 and 0.6 ± 0.3 d^-1^, respectively, were measured (Weisse, 1989). We found here consistently higher specific growth rates, especially compared with the northernmost and southernmost regions. In the tropical waters of the Sargasso Sea values were also uniformly much lower (ca 0.03 to 0.08 d^-1^, Steinberg et al., 2001). These differences are likely associated with the different methodologies being used. The BP/BB ratios used by Steinberg et al. (2001) and Grossart and Simon (2002) to estimate growth rates can be one order of magnitude lower than seawater cultures (Kirchman, 2015; but see Morán et al., 2011 for an exception). This difference is likely related to the selection of fast growing taxa in the cultures with a large impact on the growth rates estimates, while in the shorter BP incubations all bacteria are included. In his recent extensive review of bacterioplankton growth rates, Kirchman (2015) gave a mean value for heterotrophic bacteria of 1.10 ± 0.83 d^-1^ based on 26 studies using seawater cultures, slightly lower than this study (1.26 d^-1^). Recent work in the southern Bay of Biscay, using the same approach as ours, showed lower mean growth rates of all physiological groups (between 70 and 44% lower, Huete-Stauffer et al., 2015). The dominance of HNA cells, normally more active than LNA cells (Calvo-Díaz and Morán, 2006; Morán et al., 2007), together with a notably higher contribution of CTC+ bacteria, with both their absolute and relative abundances suggested as proxies of bulk bacterial production (Morán and Calvo-Díaz, 2009), in our waters point out to a higher metabolic activity of coastal Red Sea bacteria relative to their higher latitude counterparts. The high growth rates also suggest that bacteria generally thrived in a nutrient-sufficient tropical environment, in which temperature enhanced the use of available DOM.

The clear association between the carrying capacities and specific growth rates of *Live* and HNA cells suggests that the *Live* cells growing in our experiments were mainly made up of HNA bacteria, as previously observed in temperate regions (Morán et al., 2011; Huete-Stauffer et al., 2015). The significantly lower specific growth rates of LNA cells, although higher than values previously reported for the Atlantic Ocean (<0.8 d^-1^, Zubkov et al., 2001; Huete-Stauffer et al., 2015) or the Mediterranean (<0.5 d^-1^, Scharek and Latasa, 2007), confirm that LNA cells were indeed less active than the other groups (Gasol et al., 1997; Franco-Vidal and Morán, 2011). However, the overall high LNA cell growth rates could also be related to the lower presence of SAR11 bacteria within this group (Ansari et al., in prep), since SAR11 cells normally show slower growth when compared with other phylogenetic groups (Ferrera et al., 2011; Arandia-Gorostidi et al., 2017).

Although an increase in specific growth rates was measured from summer through fall (exceeding 2 d^-1^ in October 2016 for HNA and *Live* cells) (Figure 4), none of the values of the different physiological groups showed significant correlations with *in situ* temperature, suggesting that other processes can be driving the changes in growth. A similar seasonal cycle was observed at BATS station in the Sargasso Sea, where the growth rates of total bacteria peaked in July, followed by a gradual decrease until December (Steinberg et al., 2001). However, in our study the growth rates kept increasing until October, when high Chl *a* and DOC concentrations were observed (Figure 1B and Table 1). These high growth rates in periods with relatively high Chl *a* are in agreement with Huete-Stauffer et al. (2015), who also observed high growth rates during the spring phytoplankton bloom.

The amount and quality of DOM is frequently claimed to be the key limiting factor for bacterial growth (Church, 2008). The analysis of the fluorescent components of DOM suggests that variations in DOM quality indeed played an important role. Positive correlations between HNA and CTC+ specific growth rates and the consumption rates of fluorescent components C2 and C4 suggest that molecules with humic and protein nature were important to support bacterial production and respiration, respectively. These 2 components were also consistently consumed in the experiments (100 and 80% of the experiments, respectively, Figure 6). Inorganic nutrients have been also suggested to explain the variability in bacterial activity, especially phosphorus (Alonso-Sáez et al., 2007; Cuevas et al., 2011). In contrast to nitrogen, for which bacterial requirements seem to have been fulfilled with DON, since inorganic nitrate was mostly produced rather than taken up during the incubations, the consistent consumption of phosphate during the exponential phase points out to phosphorus limitation. We lack measurements of dissolved organic phosphorus (DOP), but noticeable phosphate uptake rates year-round (Table 2) indicate that DOP did not suffice to meet bacterial needs. Even if ammonium was not included, the inorganic N:P ratio was always >16, in support of strong P limitation (Downing, 1997). However, neither initial concentrations nor consumption rates of phosphate were correlated with the specific growth rates of the different bacterial groups.

The importance of heterotrophic bacteria in the pelagic food webs relies on their efficiency to produce new biomass from DOM (Sherr and Sherr, 1996; del Giorgio and Cole, 1998; Rivkin and Legendre, 2001; Carlson et al., 2007). Our measured BGE values (uniformly <13%) are in the lower range of del Giorgio and Cole (1998) review (1–64%). Robinson (2008) provided mean values of 14% for the open ocean and 19% for coastal regions. Most of the cited studies in these reviews used the “short-term approach” (i.e., using measurements of bacterial production and respiration rates separately) rather than the “long-term approach” (i.e., measurements of bacterial biomass increase and DOC consumption from the same seawater culture). According to Alonso-Sáez et al. (2008), the “short-term approach” usually yield higher BGE values. So, our slightly lower BGE values when compared with other coastal areas can then be explained by the methodology used (e.g., Reinthaler and Herndl, 2005; Alonso-Sáez et al., 2007, 2008). However, in the Mediterranean and using the same approach, Alonso-Sáez et al. (2008) also found higher BGE, that likely related with the lower Chl *a* concentrations found at our site.

Temperature and DOM lability are frequently listed as the main factors affecting BGE (Lemée et al., 2002; Carlson et al., 2007; Alonso-Sáez et al., 2008). A negative correlation between BGE and temperature was proposed by Rivkin and Legendre (2001) in their cross-system analysis. Although confirmed by other studies (Apple et al., 2006; Hall and Cotner, 2007), the role of temperature on BGE is unclear (Lemée et al., 2002; López-Urrutia and Morán, 2007; Alonso-Sáez et al., 2008). We observed a strong positive effect of *in situ* temperature on BGE, as well as that DOC consumption rates (and hence also bacterial respiration) and %DOC consumed decreased at higher temperatures. These relationships suggest that temperature may enhance the bacterial utilization of labile DOC, preferentially used to boost bacterial production rather than respiration. This assumption is also reinforced by the increase (even if not statistically significant) of C2 and C4 consumption rates at higher temperatures.

The fact that bacterial respiration was negatively correlated with *in situ* temperature is at odds with other studies (López-Urrutia, 2007; Robinson, 2008). However, other seasonal studies (e.g., Lemée et al., 2002; Alonso-Sáez et al., 2008) have failed to find significant correlations between both variables, while bacterial respiration was also negatively correlated with *in situ* temperature in the NE Atlantic (Huete-Stauffer et al., 2017), indicating that other factors could regulate respiration from a seasonal perspective. Seasonal temperature variability also covaries with bacterial community composition, DOM lability, inorganic nutrient concentrations or incident UV light, all possibly affecting bacterial metabolism. The suggestion by del Giorgio and Cole (1998) that a combination of DOM quality and inorganic nutrient availability regulate BGE was later supported by Lemée et al. (2002) and Reinthaler and Herndl (2005), with both studies showing that BGE was mainly determined by DOM lability. We believe than more important than the apparent relationship between BGE and environmental temperature is that the warmest months at our site were also those with a higher concentration of DON. This significant positive correlation of temperature with DON is likely related with the preferential bacterial remineralization of organic nitrogen over carbon from the DOM pool (Hopkinson et al., 2002).

Future studies should address how the seasonal changes in bacterioplankton community composition (Ansari et al. in prep), as shown by the changes in the physiological groups assessed here, impact biogeochemical cycles at this tropical site. In agreement with previous studies (Sarmento et al., 2010; Morán et al., 2017), our results stress the importance of considering resource availability and predation losses under future climate change scenarios. In summary, our results show that in spite of their notably low abundances, heterotrophic bacteria in shallow coastal waters of the Red Sea show high levels of activity, indicative of an adaptation to current warm conditions. Their low standing stocks should be caused by strong top-down control, at least that exerted by protistan grazers, while growth was indeed bottom-up controlled. The slightly higher allocation of assimilated organic carbon to biomass production rather to respiration was enhanced by warmer temperatures only when dissolved organic nitrogen was available.

## Author Contributions

LS led the experiment design, data analysis, and wrote the paper. LS, MC, SI, MA, and MV set up the experiments and performed the analyses. MC and TH-S contributed to the interpretation of results and writing. XM conceived the research and contributed to data analysis and interpretation and writing.

## Conflict of Interest Statement

The authors declare that the research was conducted in the absence of any commercial or financial relationships that could be construed as a potential conflict of interest.

## References

[B1] Alonso-Sáez L. Gasol J. M. Arístegui J. Vilas J. C. Vaqué D. Duarte C. M. (2007). Large-scale variability in surface bacterial carbon demand and growth efficiency in the subtropical northeast Atlantic Ocean. *Limnol. Oceanogr.* 52 533–546. 10.4319/lo.2007.52.2.0533

[B2] Alonso-Sáez L. Vázquez-Domínguez E. Cardelús C. Pinhassi J. Sala M. M. Lekunberri I. (2008). Factors controlling the year-round variability in carbon flux through bacteria in a coastal marine system. *Ecosystems* 11 397–409. 10.1007/s10021-008-9129-0

[B3] Ammerman J. W. Fuhrman J. A. Hagstrom A. Azam F. Hagström A. Azam F. (1984). Bacterioplankton growth in seawater: I. Growth kinetics and cellular characteristics in seawater cultures. *Mar. Ecol. Prog. Ser. Oldend.* 18 31–39. 10.3354/meps018031

[B4] Apple J. K. Giorgio P. A. Kemp W. M. (2006). Temperature regulation of bacterial production, respiration, and growth efficiency in a temperate salt-marsh estuary. *Aquat. Microbial. Ecol.* 43 243–254. 10.3354/ame043243

[B5] Arandia-Gorostidi N. Huete-Stauffer T. M. Alonso-Sáez L. G Morán X. A. (2017). Testing the metabolic theory of ecology with marine bacteria: different temperature sensitivity of major phylogenetic groups during the spring phytoplankton bloom. *Environ. Microbiol.* 19 4493–4505. 10.1111/1462-2920.13898 28836731

[B6] Arístegui J. Gasol J. M. Duarte C. M. Herndl G. J. (2009). Microbial oceanography of the dark oceans pelagic realm. *Limnol. Oceanogr.* 54 1501–1529. 10.4319/lo.2009.54.5.1501

[B7] Azam F. Fenchel T. Field J. G. Meyer-Reil R. A. Thingstad F. (1983). The ecological role of water column microbes in the sea. *Mar. Ecol. Prog. Ser.* 10 257–263. 10.3354/meps010257

[B8] Bird D. F. Kalff J. (1984). Empirical relationships between bacterial abundance and chlorophyll concentration in fresh and marine waters. *Can. J. Fish. Aquat. Sci.* 41 1015–1023. 10.1139/f84-118

[B9] Briand E. Pringault O. Jacquet S. Torréton J. P. (2004). The use of oxygen microprobes to measure bacterial respiration for determining bacterioplankton growth efficiency. *Limnol. Oceanogr. Methods* 2 406–416. 10.4319/lom.2004.2.406

[B10] Bunse C. Pinhassi J. (2017). Marine bacterioplankton seasonal succession dynamics. *Trends Microbiol.* 25 494–505. 10.1016/j.tim.2016.12.013 28108182

[B11] Calbet A. Agersted M. D. Kaartvedt S. Møhl M. Møller E. F. Enghoff-Poulsen S. (2015). Heterogeneous distribution of plankton within the mixed layer and its implications for bloom formation in tropical seas. *Sci. Rep.* 5:11240. 10.1038/srep11240 26062783 PMC4463021

[B12] Calleja M. L. Ansari M. I. Røstad A. Silva L. Kaartvedt S. Irigoien X. (2018). The mesopelagic scattering layer: a hotspot for heterotrophic prokaryotes in the red sea twilight Zone. *Front. Mar. Sci.* 5:259 10.3389/fmars.2018.00259

[B13] Calvo-Díaz A. Franco-Vidal L. Morán X. A. G. (2014). Annual cycles of bacterioplankton biomass and production suggest a general switch between temperature and resource control in temperate coastal ecosystems. *J. Plankton Res.* 36 859–865. 10.1093/plankt/fbu022

[B14] Calvo-Díaz A. Morán X. A. G. (2006). Seasonal dynamics of picoplankton in shelf waters of the southern Bay of Biscay. *Aquat. Microb. Ecol.* 42 159–174. 10.3354/ame042159

[B15] Carlson C. A. Del Giorgio P. A. Herndl G. J. (2007). Microbes and the dissipation of energy and respiration: from cells to ecosystems. *Oceanography* 20 89–100. 10.5670/oceanog.2007.52

[B16] Carlson C. A. Ducklow H. W. Sleeter T. D. (1996). Stocks and dynamics of bacterioplankton in the northwestern Sargasso Sea. *Deep Sea Res. Part II Top. Stud. Oceanogr.* 43 491–515. 10.1016/0967-0645(95)00101-8

[B17] Chaidez V. Dreano D. Agusti S. Duarte C. M. Hoteit I. (2017). Decadal trends in Red Sea maximum surface temperature. *Sci. Rep.* 7:8144. 10.1038/s41598-017-08146-z 28811521 PMC5557812

[B18] Church M. J. (2008). “Resource control of bacterial dynamics in the Sea,” in *Microbial Ecology of the Oceans* ed. Kirchman D. L. (Honolulu, HI: University of Hawaii at Manoa) 335–382. 10.1002/9780470281840.ch10

[B19] Church M. J. Ducklow H. W. Karl D. M. (2004). Light dependence of [3H] leucine incorporation in the oligotrophic North Pacific Ocean. *Appl. Environ. Microbiol.* 70 4079–4087. 10.1128/AEM.70.7.4079-4087.2004 15240286 PMC444811

[B20] Coble P. G. (2007). Marine optical biogeochemistry: the chemistry of ocean color. *Chem. Rev.* 107 402–418. 10.1021/cr050350+ 17256912

[B21] Cotner J. B. Biddanda B. A. (2002). Small players, large role: microbial influence on biogeochemical processes in pelagic aquatic ecosystems. *Ecosystems* 5 105–121. 10.1007/s10021-001-0059-3

[B22] Cram J. A. Chow C.-E. T. Sachdeva R. Needham D. M. Parada A. E. Steele J. A. (2015). Seasonal and interannual variability of the marine bacterioplankton community throughout the water column over ten years. *ISME J.* 9 563–580. 10.1038/ismej.2014.153 25203836 PMC4331575

[B23] Cuevas L. A. Egge J. K. Thingstad T. F. Töpper B. (2011). Organic carbon and mineral nutrient limitation of oxygen consumption, bacterial growth and efficiency in the Norwegian Sea. *Polar Biol.* 34 871–882. 10.1007/s00300-010-0944-3

[B24] del Giorgio P. A. Cole J. J. (1998). Bacterial growth efficiency in natural aquatic systems. *Annu. Rev. Ecol. Syst.* 29 503–541. 10.1146/annurev.ecolsys.29.1.503

[B25] del Giorgio P. A. Gasol J. M. (2008). Physiological structure and single-cell activity in marine bacterioplankton. *Microb. Ecol. Ocean.* 2 243–285. 10.1002/9780470281840.ch8

[B26] Downing J. A. (1997). Marine nitrogen: phosphorus stoichiometry and the global N: P cycle. *Biogeochemistry* 37 237–252. 10.1073/pnas.1423917112 26056296 PMC4500256

[B27] Ducklow H. (2000). Bacterial production and biomass in the oceans. *Microb. Ecol. Ocean.* 1 85–120.

[B28] Ducklow H. W. Carlson C. A. (1992). “Oceanic Bacterial Production,” in *Advances in Microbial Ecology* ed. Marshall K. C. (Boston, MA: Springer) 113–181. 10.1007/978-1-4684-7609-5_3

[B29] Ferrera I. Gasol J. M. Sebastián M. Hojerová E. Kobížek M. (2011). Comparison of growth rates of aerobic anoxygenic phototrophic bacteria and other bacterioplankton groups in coastal mediterranean waters. *Appl. Environ. Microbiol.* 77 7451–7458. 10.1128/AEM.00208-11 21724878 PMC3209150

[B30] Franco-Vidal L. Morán X. A. G. (2011). Relationships between coastal bacterioplankton growth rates and biomass production: comparison of leucine and thymidine uptake with single-cell physiological characteristics. *Microb. Ecol.* 61 328–341. 10.1007/s00248-010-9778-3 21120654

[B31] Fuhrman J. A. (1999). Marine viruses and their biogeochemical and ecological effects. *Nature* 399:541. 10.1038/21119 10376593

[B32] Fuhrman J. A. Hagström Å (2008). “Bacterial and archaeal community structure and its patterns,” in *Microbial Ecology of the Oceans* 2nd Edn ed. Kirchman D. L. (Hoboken, NJ: John Wiley & Sons, Inc.) 45–90. 10.1002/9780470281840.ch3

[B33] García F. C. Calleja M. L. Al-Otaibi N. Røstad A. Morán X. A. G. (2018). Diel dynamics and coupling of heterotrophic prokaryotes and dissolved organic matter in epipelagic and mesopelagic waters of the central Red Sea. *Environ. Microbiol.* 20 2990–3000. 10.1111/1462-2920.14336 30051643

[B34] Gasol J. M. del Giorgio P. A. Duarte C. M. (1997). Biomass distribution in marine planktonic communities. *Limnol. Oceanogr.* 42 1353–1363. 10.4319/lo.1997.42.6.1353

[B35] Gasol J. M. Morán X. A. G. (2015). “Flow cytometric determination of microbial abundances and its use to obtain indices of community structure and relative activity,” in *Hydrocarb. Lipid Microbiol. Protocols. Springer Protoc. Handbooks* eds McGenity T. Timmis K. Nogales B. (Berlin: Springer) 1–29. 10.1007/8623

[B36] Grégori G. Citterio S. Ghiani A. Labra M. Sgorbati S. Brown S. (2001). Resolution of viable and membrane-compromised bacteria in freshwater and marine waters based on analytical flow cytometry and nucleic acid double staining. *Appl. Environ. Microbiol.* 67 4662–4670. 10.1128/AEM.67.10.4662-4670.2001 11571170 PMC93217

[B37] Griffiths R. P. Caldwell B. A. Morita R. Y. (1984). Observations on microbial percent respiration values in arctic and subarctic marine waters and sediments. *Microb. Ecol.* 10 151–164. 10.1007/BF02011422 24221095

[B38] Grossart H. P. Simon M. (2002). Bacterioplankton dynamics in the Gulf of Aqaba and the northern Red Sea in early spring. *Mar. Ecol. Prog. Ser.* 239 263–276. 10.3354/meps239263

[B39] Gundersen K. Heldal M. Norland S. Purdie D. A. A. Knap A. H. H. (2002). Elemental C, N, and P cell content of individual bacteria collected at the Bermuda Atlantic Time-Series Study (BATS) site. *Limnol. Oceanogr.* 47 1525–1530. 10.4319/lo.2002.47.5.1525

[B40] Gundersen K. Orcutt K. M. Purdie D. A. Michaels A. F. Knap A. H. (2001). Particulate organic carbon mass distribution at the Bermuda Atlantic Time-series Study (BATS) site. *Deep. Res. Part II Top. Stud. Oceanogr.* 48 1697–1718. 10.1016/S0967-0645(00)00156-9

[B41] Hale M. S. Li W. K. W. Rivkin R. B. (2017). Meridional patterns of inorganic nutrient limitation and co-limitation of bacterial growth in the Atlantic Ocean. *Prog. Oceanogr.* 158 90–98. 10.1016/j.pocean.2016.11.007

[B42] Hall E. K. Cotner J. B. (2007). Interactive effect of temperature and resources on carbon cycling by freshwater bacterioplankton communities. *Aquat. Microb. Ecol.* 49 35–45. 10.3354/ame01124

[B43] Hopkinson C. S. Vallino J. J. Nolin A. (2002). Decomposition of dissolved organic matter from the continental margin. *Deep. Res. II* 49 4461–4478. 10.1016/S0967-0645(02)00125-X

[B44] Huete-Stauffer T. M. Arandia-Gorostidi N. Díaz-Pérez L. Morán X. A. G. (2015). Temperature dependences of growth rates and carrying capacities of marine bacteria depart from metabolic theoretical predictions. *FEMS Microbiol. Ecol.* 91 1–10. 10.1093/femsec/fiv111 26362925

[B45] Huete-Stauffer T. M. Arandia-Gorostidi N. González-Benítez N. Díaz-Pérez L. Calvo-Díaz A. Morán X. A. G. (2017). Large plankton enhance heterotrophy under experimental warming in a temperate coastal ecosystem. *Ecosystems* 21 1139–1154. 10.1007/s10021-017-0208-y

[B46] Jürgens K. Massana R. (2008). “Protistan grazing on marine bacterioplankton,” in *Microbial Ecology of the Oceans* 2nd Edn ed. Kirchman D. L. (Hoboken, NJ: Wiley) 383–441. 10.1002/9780470281840.ch11

[B47] Karl D. M. Church M. J. (2014). Microbial oceanography and the Hawaii Ocean Time-series programme. *Nat. Rev. Microbiol.* 12 699–713. 10.1038/nrmicro3333 25157695

[B48] Karner M. B. Delong E. F. Karl D. M. (2001). Archaeal dominance in the mesopelagic zone of the Pacific Ocean. *Nature* 409 507–510. 10.1038/35054051 11206545

[B49] Kirchman D. Ducklow H. Mitchell R. (1982). Estimates of bacterial growth from changes in uptake rates and biomass. *Appl. Environ. Microbiol.* 44 1296–1307. 6760812 10.1128/aem.44.6.1296-1307.1982 PMC242188

[B50] Kirchman D. L. (2008). New light on an important microbe in the ocean. *Proc. Natl. Acad. Sci. U.S.A.* 105 8487–8488. 10.1073/pnas.0804196105 18559849 PMC2438375

[B51] Kirchman D. L. (2015). Growth rates of microbes in the Oceans. *Ann. Rev. Mar. Sci.* 8 285–309. 10.1146/annurev-marine-122414-033938 26195108

[B52] Kirchman D. L. Morán X. A. G. Ducklow H. (2009). Microbial growth in the polar oceans - role of temperature and potential impact of climate change. *Nat. Rev. Microbiol.* 7 451–459. 10.1038/nrmicro2115 19421189

[B53] Kürten B. Khomayis H. S. Devassy R. Audritz S. Sommer U. Struck U. (2015). Ecohydrographic constraints on biodiversity and distribution of phytoplankton and zooplankton in coral reefs of the Red Sea, Saudi Arabia. *Mar. Ecol.* 36 1195–1214. 10.1111/maec.12224

[B54] Lemée R. Rochelle-Newall E. Van Wambeke F. Pizay M.-D. Rinaldi P. Gattuso J.-P. (2002). Seasonal variation of bacterial production, respiration and growth efficiency in the open NW Mediterranean Sea. *Aquat. Microb. Ecol.* 29 227–237. 10.3354/ame029227

[B55] Lønborg C. Martínez-García S. Teira E. Álvarez-Salgado X. A. (2011). Bacterial carbon demand and growth efficiency in a coastal upwelling system. *Aquat. Microb. Ecol.* 63 183–191. 10.3354/ame01495

[B56] Longnecker K. Wilson M. J. Sherr E. B. Sherr B. F. (2010). Effect of top-down control on cell-specific activity and diversity of active marine bacterioplankton. *Aquat. Microb. Ecol.* 58 153–165. 10.3354/ame01366

[B57] López-Urrutia Á Morán X. A. G. (2007). Resource limitation of bacterial production distorts the temperature dependence of oceanic carbon cycling. *Ecology* 88 817–822. 10.1890/06-1641 17536698

[B58] Marie D. Partensky F. Jacquet S. Vaulot D. (1997). Enumeration and cell cycle analysis of natural populations of marine picoplankton by flow cytometry using the nucleic acid stain SYBR Green I. *Appl. Environ. Microbiol.* 63 186–193. 16535483 10.1128/aem.63.1.186-193.1997 PMC1389098

[B59] McKnight D. M. Boyer E. W. Westerhoff P. K. Doran P. T. Kulbe T. Andersen D. T. (2001). Spectrofluorometric characterization of dissolved organic matter for indication of precursor organic material and aromaticity. *Limnol. Oceanogr.* 46 38–48. 10.4319/lo.2001.46.1.0038

[B60] Morán X. A. G. Alonso-Sáez L. Nogueira E. Ducklow H. W. González N. López-Urrutia Á (2015). More, smaller bacteria in response to ocean’s warming? *Proc. R. Soc. B Biol. Sci.* 282:20150371. 10.1098/rspb.2015.0371 26063843 PMC4590472

[B61] Morán X. A. G. Bode A. Suárez L. Á Nogueira E. (2007). Assessing the relevance of nucleic acid content as an indicator of marine bacterial activity. *Aquat. Microb. Ecol.* 46 141–152. 10.3354/ame046141

[B62] Morán X. A. G. Calvo-Díaz A. (2009). Single-cell vs. bulk activity properties of coastal bacterioplankton over an annual cycle in a temperate ecosystem. *FEMS Microbiol. Ecol.* 67 43–56. 10.1111/j.1574-6941.2008.00601.x 19120458

[B63] Morán X. A. G. Calvo-Díaz A. Arandia-Gorostidi N. Huete-Stauffer T. M. (2018). Temperature sensitivities of microbial plankton net growth rates are seasonally coherent and linked to nutrient availability. *Environ. Microbiol.* 20 3798–3810. 10.1111/1462-2920.14393 30159999

[B64] Morán X. A. G. Calvo-Díaz A. Ducklow H. W. (2010). Total and phytoplankton mediated bottom-up control of bacterioplankton change with temperature in NE Atlantic shelf waters. *Aquat. Microb. Ecol.* 58 229–239. 10.3354/ame01374

[B65] Morán X. A. G. Ducklow H. W. Erickson M. (2011). Single-cell physiological structure and growth rates of heterotrophic bacteria in a temperate estuary (Waquoit Bay, Massachusetts). *Limnol. Oceanogr.* 56 37–48. 10.4319/lo.2011.56.1.0037

[B66] Morán X. A. G. Estrada M. Gasol J. M. Pedrós-Alió C. (2002). Dissolved primary production and the strength of phytoplankton-bacterioplankton coupling in contrasting marine regions. *Microb. Ecol.* 44 217–223. 10.1007/s00248-002-1026-z 12209254

[B67] Morán X. A. G. Gasol J. M. Pernice M. C. Mangot J. F. Massana R. Lara E. (2017). Temperature regulation of marine heterotrophic prokaryotes increases latitudinally as a breach between bottom-up and top-down controls. *Glob. Chang. Biol.* 23 3956–3964. 10.1111/gcb.13730 28423463

[B68] Murphy K. R. Butler K. D. Spencer R. G. M. Stedmon C. A. Boehme J. R. Aiken G. R. (2010). Measurement of dissolved organic matter fluorescence in aquatic environments: an interlaboratory comparison. *Environ. Sci. Technol.* 44 9405–9412. 10.1021/es102362t 21069954

[B69] Murphy K. R. Stedmon C. A. Graeber D. Bro R. (2013). Fluorescence spectroscopy and multi-way techniques. PARAFAC. *Anal. Methods* 5 6557–6566. 10.1039/c3ay41160e

[B70] Ngugi D. K. Antunes A. Brune A. Stingl U. (2012). Biogeography of pelagic bacterioplankton across an antagonistic temperature-salinity gradient in the Red Sea. *Mol. Ecol.* 21 388–405. 10.1111/j.1365-294X.2011.05378.x 22133021

[B71] Pearman J. K. Ellis J. Irigoien X. Sarma Y. V. B. Jones B. H. Carvalho S. (2017). Microbial planktonic communities in the Red Sea: high levels of spatial and temporal variability shaped by nutrient availability and turbulence. *Sci. Rep.* 7 1–15. 10.1038/s41598-017-06928-z 28747798 PMC5529573

[B72] Rasul N. M. A. Stewart I. C. F. Nawab Z. A. (2015). “Introduction to the Red Sea: its origin, structure, and environment,” in *The Red Sea. Springer Earth System Sciences* eds Rasul N. Stewart I. (Berlin: Springer) 1–28. 10.1007/978-3-662-45201-1_1

[B73] Reinthaler T. Herndl G. (2005). Seasonal dynamics of bacterial growth efficiencies in relation to phytoplankton in the southern North Sea. *Aquat. Microb. Ecol.* 39 7–16. 10.3354/ame039007

[B74] Rivkin R. B. Legendre L. (2001). Biogenic carbon cycling in the upper ocean: Effects of microbial respiration. *Science* 291 2398–2400. 10.1126/science.291.5512.2398 11264533

[B75] Robinson C. (2008). “Heterotrophic bacterial respiration,” in *Microbial Ecology of the Oceans* 2nd Edn ed. Kirchman D. L. (New York, NY: Wiley-Liss) 299–334. 10.1002/9780470281840.ch9

[B76] Ruiz-González C. Galí M. Lefort T. Cardelús C. Simó R. Gasol J. M. (2012). Annual variability in light modulation of bacterial heterotrophic activity in surface northwestern Mediterranean waters. *Limnol. Oceanogr.* 57 1376–1388. 10.4319/lo.2012.57.5.1376

[B77] Sarmento H. Montoya J. M. Vazquez-Dominguez E. Vaque D. Gasol J. M. (2010). Warming effects on marine microbial food web processes: how far can we go when it comes to predictions? *Philos. Trans. R. Soc. B Biol. Sci.* 365 2137–2149. 10.1098/rstb.2010.0045 20513721 PMC2880134

[B78] Scharek R. Latasa M. (2007). Growth, grazing and carbon flux of high and low nucleic acid bacteria differ in surface and deep chlorophyll maximum layers in the NW Mediterranean Sea. *Aquat. Microb. Ecol.* 46 153–161. 10.3354/ame046153

[B79] Sherr B. F. Del Giorgio P. Sherr E. B. (1999). Estimating abundance and single-cell characteristics of respiring bacteria via the redox dye CTC. *Aquat. Microb. Ecol.* 18 117–131. 10.3354/ame018117

[B80] Sherr E. B. Sherr B. F. (1996). Temporal offset in oceanic production and respiration processes implied by seasonal changes in atmospheric oxygen: the role of heterotrophic microbes. *Aquat. Microb. Ecol.* 11 91–100. 10.3354/ame011091

[B81] Stedmon C. A. Bro R. (2008). Characterizing dissolved organic matter fluorescence with parallel factor analysis: a tutorial. *Limnol. Oceanogr. Methods* 6 572–579. 10.4319/lom.2008.6.572

[B82] Stedmon C. A. Markager S. Bro R. (2003). Tracing dissolved organic matter in aquatic environments using a new approach to fluorescence spectroscopy. *Mar. Chem.* 82 239–254. 10.1021/acs.est.8b02648 30157380

[B83] Steinberg D. K. Carlson C. A. Bates N. R. Johnson R. J. Michaels A. F. Knap A. H. (2001). Overview of the US JGOFS Bermuda Atlantic Time-series Study (BATS): a decade-scale look at ocean biology and biogeochemistry. *Deep. Res. Part II Top. Stud. Oceanogr.* 48 1405–1447. 10.1016/S0967-0645(00)00148-X

[B84] Tesfamichael D. Pauly D. (2016). *The Red Sea Ecosystem and Fisheries.* Berlin: Springer 10.1007/978-94-017-7435-2

[B85] Thingstad T. F. (2000). “Control of bacterial growth in idealized food webs,” in *Microbial Ecology of the Oceans* ed. Kirchman D. L. (New York, NY: Wiley) 229–260. 10.1371/journal.pone.0101415

[B86] Thingstad T. F. Lignell R. (1997). Theoretical models for the control of bacterial growth rate, abundance, diversity and carbon demand. *Aquat. Microb. Ecol.* 13 19–27. 10.3354/ame013019

[B87] Thompson L. R. Williams G. J. Haroon M. F. Shibl A. Larsen P. Shorenstein J. (2017). Metagenomic covariation along densely sampled environmental gradients in the Red Sea. *ISME J.* 11 138–151. 10.1038/ismej.2016.99 27420030 PMC5315489

[B88] Vaqué D. Alonso-Sáez L. Arístegui J. Agustí S. Duarte C. M. Montserrat Sala M. (2014). Bacterial production and losses to predators along an open ocean productivity gradient in the Subtropical North East Atlantic Ocean. *J. Plankton Res.* 36 198–213. 10.1093/plankt/fbt085

[B89] Vrede K. Heldal M. Norland S. Bratbak G. (2002). Elemental composition (C, N, P) and cell volume of exponentially growing and nutrient-limited bacterioplankton. *Appl. Environ. Microbiol.* 68 2965–2971. 10.1128/AEM.68.6.2965-2971.2002 12039756 PMC123973

[B90] Weisse T. (1989). The microbial loop in the Red Sea. dynamics of pelagic bacteria and heterotrophic nanoflagellates. *Mar. Ecol. Prog. Ser.* 55 241–250. 10.3354/meps055241

[B91] Wetz M. S. Wheeler P. A. (2004). Response of bacteria to simulated upwelling phytoplankton blooms. *Mar. Ecol. Prog. Ser.* 272 49–57. 10.3354/meps272049

[B92] White P. A. Kalff J. Rasmussen J. B. Gasol J. M. (1991). The effect of temperature and algal biomass on bacterial production and specific growth rate in freshwater and marine habitats. *Microb. Ecol.* 21 99–118. 10.1007/BF02539147 24194204

[B93] Wiebinga C. J. Veldhuis M. J. W. De Baar H. J. W. (1997). Abundance and productivity of bacterioplankton in relation to seasonal upwelling in the northwest Indian Ocean. *Deep. Res. Part I Oceanogr. Res. Pap.* 44 451–476. 10.1016/S0967-0637(96)00115-X

[B94] Worden A. Z. Nolan J. K. Palenik B. (2004). Assessing the dynamics and ecology of marine picophytoplankton: the importance of the eukaryotic component. *Limnol. Oceanogr.* 49 168–179. 10.4319/lo.2004.49.1.0168

[B95] Zubkov M. V. Fuchs B. M. Burkill P. H. Amann R. (2001). Comparison of cellular and biomass specific activities of dominant bacterioplankton groups in stratified waters of the celtic Sea. *Appl. Environ. Microbiol.* 67 5210–5218. 10.1128/AEM.67.11.5210-5218.2001 11679347 PMC93292

